# Capacity Evaluation of Diagnostic Tests For COVID-19 Using Multicriteria Decision-Making Techniques

**DOI:** 10.1155/2020/1560250

**Published:** 2020-08-06

**Authors:** Murat Sayan, Figen Sarigul Yildirim, Tamer Sanlidag, Berna Uzun, Dilber Uzun Ozsahin, Ilker Ozsahin

**Affiliations:** ^1^Faculty of Medicine, Clinical Laboratory, PCR Unit, Kocaeli University, Kocaeli, Turkey; ^2^DESAM Institute, Near East University, Nicosia/TRNC, Mersin-10, 99138, Turkey; ^3^Health Science University, Antalya Education and Research Hospital, Department of Infectious Diseases and Clinical Microbiology, Antalya 07050, Turkey; ^4^Department of Medical Microbiology, Manisa Celal Bayar University, Manisa, Turkey; ^5^Department of Mathematics, Near East University, Nicosia/TRNC, Mersin-10, 99138, Turkey; ^6^Department of Biomedical Engineering, Faculty of Engineering, Near East University, Nicosia/TRNC, Mersin-10, 99138, Turkey

## Abstract

In December 2019, cases of pneumonia were detected in Wuhan, China, which were caused by the highly contagious coronavirus. This study is aimed at comparing the confusion regarding the selection of effective diagnostic methods to make a mutual comparison among existing SARS-CoV-2 diagnostic tests and at determining the most effective one. Based on available published evidence and clinical practice, diagnostic tests of coronavirus disease (COVID-19) were evaluated by multi-criteria decision-making (MCDM) methods, namely, fuzzy preference ranking organization method for enrichment evaluation (fuzzy PROMETHEE) and fuzzy technique for order of preference by similarity to ideal solution (fuzzy TOPSIS). Computerized tomography of chest (chest CT), the detection of viral nucleic acid by polymerase chain reaction, cell culture, CoV-19 antigen detection, CoV-19 antibody IgM, CoV-19 antibody IgG, and chest X-ray were evaluated by linguistic fuzzy scale to compare among the diagnostic tests. This scale consists of selected parameters that possessed different weights which were determined by the experts' opinions of the field. The results of our study with both proposed MCDM methods indicated that the most effective diagnosis method of COVID-19 was chest CT. It is interesting to note that the methods that are consistently used in the diagnosis of viral diseases were ranked in second place for the diagnosis of COVID-19. However, each country should use appropriate diagnostic solutions according to its own resources. Our findings also show which diagnostic systems can be used in combination.

## 1. Introduction

After cases of pneumonia of unknown cause were detected in Wuhan, China, in December 2019, a new coronavirus was isolated from human airway epithelial cells and was named severe acute respiratory syndrome coronavirus 2 (SARS-CoV-2), which is responsible for coronavirus disease (COVID-19) [1]. SARS-CoV-2 is also a member of the coronavirus family that includes Middle East Respiratory Syndrome- (MERS-) CoV and SARS-CoV, which infect humans [1, 2]. Wild animals are the source of the infection.

According to phylogenetic analysis of full genome sequencing, the coronavirus that causes COVID-19 is a betacoronavirus in the same subgenus clade as SARS-CoV-2. The structure of the receptor binding site for cell entry is similar and uses the angiotensin-converting enzyme 2 receptor found in the epithelial cells of the alveoli used by SARS-CoV-2 [3]. The International Committee on Taxonomy of Viruses has proposed that this virus be designated as SARS-CoV-2 [3, 4].

The main mode of transmission is via person-to-person spread. When an infected person coughs, sneezes, or speaks, the virus released in respiratory secretions can infect another person if it comes into direct contact with the mucous membranes through droplet delivery. Also, infection can occur if a person touches an infected surface and then their eyes, nose, or mouth [5]. Infected but asymptomatic people can transmit the virus to others [6].

The most common serious sign of infection is pneumonia: it is characterized by fever, cough, shortness of breath, and bilateral infiltrates in chest imaging [7–9]. In severe cases, patients can quickly experience acute respiratory syndrome, septic shock, metabolic acidosis, and coagulopathy [10, 11]. The mortality rate is 1-2%, but this rate may increase up to 14%, especially in elderly patients with comorbidities such as hypertension, diabetes mellitus, or cardiovascular diseases [10]. Due to the strong infectivity of SARS-CoV-2, it is necessary to identify, isolate, and treat patients as soon as possible, which can reduce mortality rates while reducing the risk of public contamination. In order to be able to treat patients, it is imperative that the disease is diagnosed quickly and accurately. The diagnosis is based on real-time reverse transcription-polymerase chain reaction (rRT-PCR) positivity for the presence of coronavirus [12]. With nucleic acid isolation processing, rRT-PCR results usually require 5 to 6 hours. In addition, it remains unclear whether rRT-PCR is the gold standard and whether false-positive or false-negative results are common. The Centers for Disease Control and Prevention recommends that nasopharyngeal and oropharyngeal swab specimens should be collected to test for SARS-CoV-2 [13]. Although false-positive tests are generally possible, a positive test for SARS-CoV-2 confirms the diagnosis of COVID-19. False-negative results can be obtained from the upper respiratory samples, so if the first test is negative and the patient continues to suspect COVID-19, it is recommended that the test be repeated [14]. Serological tests, on the other hand, can be accessed and evaluated more easily and can identify patients with existing or previous infections but who have negative rRT-PCR tests [15]. These tests can also be used because they are easier to access in places that do not have access to the rRT-PCR test. The technique of the test is quite simple. It gives results in a short time [16]. Although samples taken from the patient are not evaluated for viral culture, they do have diagnostic value [17].

The other methods that are used in the diagnosis of COVID-19 are imaging methods. The diagnostic sensitivity of viral pneumonia by chest radiography is relatively low [18], whereas computerized tomography (CT) has high sensitivity for diagnosis of COVID-19 which makes it a primary tool for COVID-19 detection in epidemic areas [19]. In COVID-19 patients, the sensitivity of chest CT scan is 97% but its specificity is 25% with rRT-PCR results as reference, which is why it is not used as a primary screening test [19].

Chest CT scan abnormalities have also been identified in patients prior to the development of symptoms and even prior to the detection of viral RNA from upper respiratory specimens [20, 21]. In the article published by Huang et al. in the *Lancet*, it was stated that it is necessary to have signs of infection and/or a positive nucleic acid test for chest CT scan to be used in diagnosis; otherwise, CT should not be recommended for screening or early diagnosis [22]. They said that CT should not be recommended for screening or early diagnosis. However, when the rRT-PCR results of patients were considered to be clinically and epidemiologically negative for COVID-19, chest CT scan became more valuable than rRT-PCR in the early stage of the disease. Shi et al. showed that combining the evaluation of chest CT scan imaging features with clinical and laboratory findings may facilitate the early diagnosis of COVID-19 pneumonia [20, 23].

Thus far, seven different diagnostic tests have been used for COVID-19: rRT- PCR, cell culture, CoV-19 antigen detection, serological tests (CoV-19 antibody IgM, CoV-19 antibody IgG), chest X-ray, and chest CT. From the onset of the pandemic, real-life experience has shown that rRT-PCR and chest CT are the most preferred methods for the diagnosis of COVID-19. The suitability of SARS-CoV-2 diagnostic tests can be prioritized with the parameters of high sensitivity, high specificity, low false positivity, low false negativity, high usability, low cost, etc. In this study, we used the fuzzy preference ranking organization method for enrichment evaluation (fuzzy PROMETHEE) and fuzzy technique for order of preference by similarity to ideal solution (fuzzy TOPSIS) techniques to compare the confusion regarding the selection of effective diagnostic methods to make a mutual comparison between the aforementioned seven SARS-CoV-2 diagnostic tests and hence determine the best one. We have decided to use fuzzy PROMETHEE because fuzzy part can handle fuzzy data very well, and PROMETHEE part can handle the data when there are too many parameters to be set properly. One other advantage of using PROMETHEE is that it is a user-friendly outranking method and successfully adapted to real-life problems. Fuzzy TOPSIS, on the other hand, is simple, rational, and comprehensive, as well as able to measure relative performance of alternatives.

## 2. Materials and Methods

Fuzzy PROMETHEE and fuzzy TOPSIS techniques, which are widely used by researchers as MCDM methods, were applied for the evaluation of the SARS-CoV-2 diagnostic tests in order to provide the best alternatives. The fuzzy PROMETHEE technique is a hybrid model that provides decision analysis for nonnumerical data and also enables decision-makers to define vague conditions mathematically [24]. The PROMETHEE technique was developed by Brans et al. [25, 26], and it is aimed at giving a comprehensive ranking for a finite set of alternatives corresponding to their criteria and the importance level of each criterion. By providing various preference functions, including V-shaped function, level function, Gaussian function, U-shaped function, and linear function, to the criteria for the comparison of the alternatives, the PROMETHEE technique differs from the other MCDM methods and can provide more sensitive results. The PROMETHEE and TOPSIS methods are unable to analyze fuzzy data (linguistic or vague data) in the actual decision-making environment. Thus, we have applied the fuzzy-based PROMETHEE and TOPSIS techniques, which are the hybrid methods that use a fuzzy scale. The fuzzy scale supports the decision-maker in decision analysis if the data is within a range, vague, or qualitative.

In this study, a linguistic fuzzy scale has been used to define the parameters of the SARS-CoV-2 diagnostic tests. These values were converted to triangular fuzzy numbers, and then the Yager index was applied for defuzzification of the data. Then, the PROMETHEE decision lab program with a Gaussian preference function was applied for the determination of the net ranking result.

The PROMETHEE method comprises 5 steps that are applied for the MCDM analysis and to rank the alternatives as follows [25–28]:

(i) The preference function *P*_*j*_ of each criterion *j* should be defined
(ii) Importance weights of each criterion *w*_*j*_ = (*w*_1_, *w*_2_, ⋯, *w*_*K*_), where (*j* = 1, 2, ⋯, *K*) should be defined as
  (1) $$\begin{array}{c} \overset{K}{\underset{j=1}{\sum }}w_{j}=1, \end{array}$$
  where *K* is the number of criteria.
(iii) For each of the alternative pairs *a*_*t*_, *a*_*t*′_ ∈ *A*, the outranking relation *π*(*a*_*t*_, *a*_*t*′_) should be determined by
  (2) $$\begin{array}{c} \pi \left(a_{t},a_{t^{\prime }}\right)=\overset{K}{\underset{j=1}{\sum }}w_{j}.\left[P_{j}\left(f_{j}\left(a_{t}\right)-f_{j}\left(a_{t^{\prime }}\right)\right)\right],\;AXA⟶\left[0,1\right], \end{array}$$
  where *f*_*j*_(*a*_*t*_) denotes the value of the *j*^th^ criterion of the alternative *a*_*t*_ and *π*(*a*_*t*_, *a*_*t*′_) denotes the preference indices, which show the preference intensity for an alternative *a*_*t*_ in comparison to an alternative *a*_*t*′_ while counting all criteria simultaneously.
(iv) The positive outranking flow and negative outranking flow should be determined as follows:
  (a) A positive outranking flow of the alternative *a*_*t*_:
  (3) $$\begin{array}{c} \Phi^{+}\left(a_{t}\right)=\frac{1}{n-1}\underset{t^{\prime }\neq t}{\overset{n}{\underset{t^{\prime }=1}{\sum }}}\pi \left(a_{t},a_{t^{\prime }}\right). \end{array}$$
  (b) A negative outranking flow of the alternative *a*_*t*_:
    (4) $$\begin{array}{c} \Phi^{-}\left(a_{t}\right)=\frac{1}{n-1}\underset{t^{\prime }\neq t}{\overset{n}{\underset{t^{\prime }=1}{\sum }}}\pi \left(a_{t^{\prime }},a_{t}\right). \end{array}$$
    *n* denotes the number of alternatives. The *Φ*^+^(*a*_*t*_)  defines the strength of alternative *a*_*t*_ ∈ A, while the negative outranking flow *Φ*^−^(*a*_*t*_) defines the weakness of alternative *a*_*t*_ ∈ A.

PROMETHEE I determines a partial preorder of the alternatives based on the positive and negative outranking flows, and PROMETHEE II determines a complete preorder of the alternatives based on a net flow. The partial preorder of the options can be determined based on the following statements:

Via PROMETHEE I, alternative *a*_*t*_ is selected to alternative *a*_*t*′_(*a*_*t*_*Pa*_*t*′_) if it satisfies either of the statements given below as

(5) $$\begin{array}{c} \left\{\begin{array}{c} \Phi^{+}\left(a_{t}\right)\geq \Phi^{+}\left(a_{t^{\prime }}\right)\text{ and }\Phi^{-}\left(a_{t}\right)<\Phi^{-}\left(a_{t^{\prime }}\right),\\ \Phi^{+}\left(a_{t}\right)>\Phi^{+}\left(a_{t^{\prime }}\right)\text{ and }\Phi^{-}\left(a_{t}\right)=\Phi^{-}\left(a_{t^{\prime }}\right). \end{array}\right. \end{array}$$

*a*_*t*_ is indifferent to alternative *a*_*t*′_(*a*_*t*_*Ia*_*t*′_) if

(6) $$\begin{array}{c} \Phi^{+}\left(a_{t}\right)=\Phi^{+}\left(a_{t^{\prime }}\right)\text{ and }\Phi^{-}\left(a_{t}\right)=\Phi^{-}\left(a_{t^{\prime }}\right). \end{array}$$

And *a*_*t*_ is incomparable to *a*_*t*′_(*a*_*t*_*Ra*_*t*′_) if

(7) $$\begin{array}{c} \left\{\begin{array}{c} \Phi^{+}\left(a_{t}\right)>\Phi^{+}\left(a_{t^{\prime }}\right)\text{ and }\Phi^{-}\left(a_{t}\right)>\Phi^{-}\left(a_{t^{\prime }}\right),\\ \Phi^{+}\left(a_{t}\right)<\Phi^{+}\left(a_{t^{\prime }}\right)\text{ and }\Phi^{-}\left(a_{t}\right)<\Phi^{-}\left(a_{t^{\prime }}\right). \end{array}\right. \end{array}$$

The net outranking flow can be calculated for each alternative by using the following:

(8) $$\begin{array}{c} \Phi^{\text{net}}\left(a_{t}\right)=\Phi^{+}\left(a_{t}\right)-\Phi^{-}\left(a_{t}\right). \end{array}$$

Via PROMETHEE II, the complete order with net flow can be determined as

(9) $$\begin{array}{c} a_{t}\text{ is preferred to }a_{t^{\prime }}\;\left(a_{t}Pa_{t^{\prime }}\right)\;\text{if }\Phi^{\text{net}}\left(a_{t}\right)>\Phi^{\text{net}}\left(a_{t^{\prime }}\right),\\ a_{t}\text{ is indifferent to }a_{t^{\prime }}\;\left(a_{t}Ia_{t^{\prime }}\right)\;\text{if }\Phi^{\text{net}}\left(a_{t}\right)=\Phi^{\text{net}}\left(a_{t^{\prime }}\right). \end{array}$$

The higher *Φ*^net^(*a*_*t*_) value provides the better alternative.

In this study, triangular fuzzy scale was used for defining the linguistic data of the criteria of SARS-CoV-2 diagnostic tests as shown in Table 1. Zadeh [29] has defined the fuzzy sets in order to define the linguistic or vague data mathematically based on the membership degree. Triangular fuzzy sets can be represented with 3 specific points (*a*, *b*, and *c*) where *a* is the initial point (left bound), *b* is the peak point of the triangle which contain the maximum membership degree, and *c* is the right bound of the triangle. The linguistic fuzzy scale indicates the linguistic terms of very high, high, medium, and low with their associated fuzzy sets. Furthermore, the importance level of each has been defined with the triangular fuzzy scale by the experts for analyzing the effectiveness criteria of SARS-CoV-2 diagnostic tests. The criteria selected for the analysis include cost, accessibility, sensitivity, and specificity.

For the validation of the fuzzy-based PROMETHEE output, fuzzy TOPSIS, a different type of MCDM technique, has been used for the same dataset. TOPSIS method was first proposed by Yoon and Hwang [30] and commonly applied for the decision-making problems of the conflicting criteria [31, 32] in wide disciplines. It compares a set of alternatives by identifying weights for each criterion, normalizing scores for each criterion and calculating the geometric distance between each alternative and the ideal alternative, which is the best score in each criterion. It based on the concept that the chosen alternative should have the shortest geometric distance from the positive ideal solution (PIS) and the longest geometric distance from the negative ideal solution (NIS).

There are 6 steps of the TOPSIS method to be applied for the MCDM analysis and rank the alternatives as follows [31].

(1) The decision matrix which includes the parameters (criteria) of the alternatives and the importance weight of the criteria, *j*(*w*_*j*_), should be defined as
  (10) $$\begin{array}{c} \overset{K}{\underset{j=1}{\sum }}w_{j}=1, \end{array}$$
  where *K* is the number of criteria.
(2) The normalized decision matrix (*n*_*ij*_) should be calculated by one of the equations below:
  (11) $$\begin{array}{c} n_{ij}=\frac{X_{ij}}{\sqrt{\sum_{i=1}^{m}X_{ij}^{2}}},\\ n_{ij}=\frac{X_{ij}}{\underset{i}{\max}X_{ij}},\\ n_{ij}=\left\{\begin{array}{c} \frac{x_{ij}-\underset{i}{\min}x_{ij}}{\underset{i}{\max}x_{ij}-\underset{i}{\min}x_{ij}},\\ \frac{\underset{i}{\max}x_{ij}-x_{ij}}{\underset{\text{i}}{\max}x_{ij}-\underset{i}{\min}x_{ij}}, \end{array}\right. \end{array}$$
  where *x*_*ij*_ is the value of the *i*^th^ alternative and *j*^th^ criterion.
(3) The values of the weighted normalized matrix ( *v*_*ij*_) should be calculated by applying the following:
  (12) $$\begin{array}{c} \;v_{ij}=w_{j}n_{ij}. \end{array}$$
(4) And positive ideal solution (*A*^+^) and negative ideal solution (*A*^−^)  should be determined by using Equation (13) and Equation (14), respectively
  (13) $$\begin{array}{c} A^{+}=\left(v_{1}^{+},v_{2}^{+},\cdots .,v_{n}^{+}\right)=\left[\left[\underset{i}{\max}v_{ij}\left|j\in I\right.\right],\left[\underset{i}{\min}v_{ij}\left|j\in J\right.\right]\right], \end{array}$$
  (14) $$\begin{array}{c} A^{-}=\left(v_{1}^{-},v_{2}^{-},\cdots .,v_{n}^{-}\right)=\left[\left[\underset{i}{\min}v_{ij}\left|j\in I\right.\right],\left[\underset{i}{\max}v_{ij}\left|j\in J\right.\right]\right], \end{array}$$
  where *I* and *J* are associated with benefit criteria and cost criteria, for *i* = 1, ⋯, *m*; *j* = 1, ⋯, *n*.
 Positive ideal solution (*A*^+^) is the set of the values that maximizes the benefit criteria while minimizing the cost criteria. Negative ideal solution (*A*^−^) can be considered an opposite of the positive ideal solution. In the negative ideal solution, the benefit criterion is minimized while the cost criterion is maximized. According to the TOPSIS method, the most suitable option is the one which is closer to the positive ideal solution and further to the negative ideal solution [33].
(5) Then, the separation measures between alternatives and the positive ideal solution should be calculated by using Equation (15) and the separation measures between alternatives and the negative ideal solution should be calculated by using Equation (16) in order to obtain the distance between the *i*^th^ alternative and the positive ideal solution (*d*_*i*_^+^) and the distance between the *i*^th^ alternative and the negative ideal solution (*d*_*i*_^−^) as shown below:
  (15) $$\begin{array}{c} d_{i}^{+}={\left(\overset{n}{\underset{j=1}{\sum }}{\left(v_{ij}-v_{j}^{+}\right)}^{p}\right)}^{1/p},\;i=1,2,\cdots ,m, \end{array}$$
  (16) $$\begin{array}{c} d_{i}^{-}={\left(\overset{n}{\underset{j=1}{\sum }}{\left(v_{ij}-v_{j}^{-}\right)}^{p}\right)}^{1/p},\;i=1,2,\cdots ,m, \end{array}$$
  where *p* ≥ 1.
(6) Then, the relative closeness to the positive ideal solution (*R*_*i*_) should be calculated for every alternative by using Equation (17) as seen below:
  (17) $$\begin{array}{c} R_{i}=\frac{d_{i}^{-}}{d_{i}^{-}+d_{i}^{+}}, \end{array}$$
  where 0 ≤ *R*_*i*_ ≤ 1, *i* = 1, 2, ⋯.., *m*.

Positive and negative ideal solution sets of the SARS-CoV-2 diagnostic tests have been obtained as shown in Table 2 below.

In TOPSIS technique, relative closeness to the positive ideal solution (*R*_*i*_) determines the ranking results of the alternatives. The higher (*R*_*i*_) is a more preferred option. The weighted normalized dataset of the SARS-CoV-2 diagnostic tests can be seen in Table 3.

## 3. Results and Discussion

Table 4 indicates the results of the complete ranking for the SARS-CoV-2 diagnostic tests, where each alternative pair is compared numerically based on each criterion and their importance weights. The power of each alternative can be thought numerically as the positive outranking flow, while the weakness of the alternatives can be thought as the negative outranking flow. However, the net flow gives the net ranking results. The most effective alternative is the one with the higher net flow. With the maximum positive outranking flow and minimum negative outranking flow, the chest CT is the best available SARS-CoV-2 diagnostic test, followed by naso/oropharyngeal swab rRT-PCR. Even if naso/oropharyngeal swab rRT-PCR has a lower positive flow than cell culture, it also has lower negative flow than the cell culture. Therefore, the second-best option is naso/oropharyngeal swab rRT-PCR with 0.0258 net flow, and the third one is the cell culture with the 0.0098 net flow. The last two options are CoV-19 antigen detection and chest X-ray as seen in Table 4.

**Figure 1:**
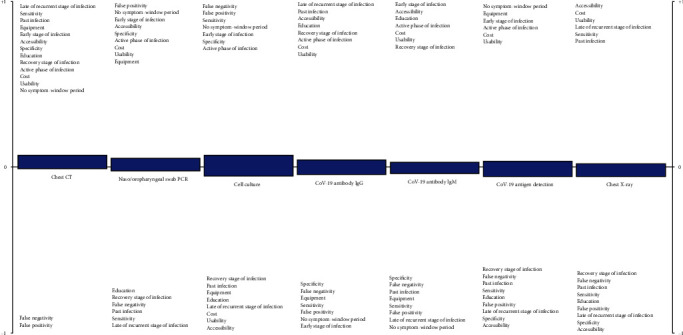
Positive and negative aspects of each technique obtained by fuzzy PROMETHEE.

The strength and the weakness of the available SARS-CoV-2 diagnostic tests are shown in Figure 1. The criteria for each SARS-CoV-2 diagnostic tests are listed above or below the zero-threshold level based on their effect on the performance of the alternatives. If the criteria are in advantage of the technique, they are listed above, and if the criteria are in disadvantage of the technique, they are listed below the zero-threshold level. As represented, the most preferred technique is the chest CT, with almost all the criteria listed above the threshold level, while the last effective technique for the SARS-CoV-2 diagnosis is the chest X-ray, with most of the criteria listed above the threshold level. These results can be useful for hospitals, doctors, governments, and patients or anyone involved in the decision-making processes of the SARS-CoV-2 diagnosis.

The SARS-CoV-2 diagnostic tests ranking results obtained with fuzzy TOPSIS have been presented in Table 5. With the 0.6403 value of the closeness to the ideal solution (*R*_*i*_), chest CT is the best alternative, naso/oropharyngeal swab rRT-PCR is the second best alternative with 0.5865 *R*_*i*_, and the cell culture with 0.5718  *R*_*i*_ is the third best option between the alternatives. The CoV-19 antigen detection and chest X-ray were found as the least effective options.

These results are consistently similar with the fuzzy-based PROMETHEE outputs. Hence, the results from both techniques could give evidence to the decision-makers about the effectiveness of the available SARS-CoV-2 diagnostic tests.

According to the findings of a study conducted with more than 1,000 patients by Ai et al. [19], CT should be the primary diagnosis tool used to screen for COVID-19. However, their finding of 97% sensitivity for the chest CT was based on positive rRT-PCR test results. Chest CT scan for COVID-19 diagnosis can be a good complement to rRT-PCR [34]. According to the study of Fang et al. [34], the sensitivity of CT for COVID-19 was higher compared to rRT-PCR (98% vs. 71%, respectively). Similarly, we also showed mathematically that CT should be the first preferred diagnostic test. Furthermore, differentiating COVID-19 from non-COVID-19 pneumonia on chest CT, which is a relatively harder task, was studied in [35], and the authors reported that the findings of CT are considered nonspecific despite its high sensitivity. In addition, one of the greatest issues faced is the need for an expert radiologist to interpret the radiography images. This procedure is even more difficult when attempting to differentiate COVID-19 from other types of pneumonia since the visual indicators can be subtle [35].

Therefore, the main limitation of our study is the lack of analysis of the combination of different diagnostic tests. Hence, in future studies, once the available techniques for diagnosing COVID-19 are more robust, we will apply the MCDM methods to the combination of different diagnostic tests. In addition, it is sensible to apply the MCDM methods for specific countries, regions, or even hospitals considering specific conditions such as their available diagnostic toolkits, scanners, and experts.

## 4. Conclusions

This study analyzed the seven selected available COVID-19 diagnostic tests using the fuzzy-based MCDM methods. The evaluations of the effectiveness of the alternatives were made based on the selected criteria, and their corresponding weights were determined by the experts. As a result of the evaluation, chest CT was found to be the most effective diagnostic test. Moreover, the results indicated that the top three most effective COVID-19 diagnostic tests are chest CT, rRT-PCR, and cell culture. On the other hand, chest X-ray is the last effective diagnostic test. It is interesting to note that the methods that are always used in the diagnosis of viral diseases were ranked second for the diagnosis of COVID-19.

In this study, during the process of evaluating the effectiveness of the given COVID-19 diagnostic tests, each technique was considered and compared based on their general properties collected from the latest available guidelines. The use of fuzzy-based MCDM methods can be also adapted based on the patient's specific condition.

## Figures and Tables

**Table 1:** Linguistic fuzzy scale.

Linguistic scale for evaluation	Triangular fuzzy scale	Importance ratings of criteria
VH	(0.75, 1, 1)	No symptom-window period, early stage of infection, active phase of infection, late of recurrent stage of infection, cost, accessibility, false positivity, false negativity
H	(0.50, 0.75, 1)	Sensitivity, specificity
M	(0.25, 0.50, 0.75)	Past infection, usability, equipment, education
L	(0, 0.25, 0.50)	Recovery stage of infection
VL	(0, 0, 0.25)	

VH: very high; H: high; M: medium; L: low; VL: very low.

**Table 2:** Positive and negative ideal solution sets.

Criteria	Positive ideal solution	Negative ideal solution
No symptom-window period	0.473	0.158
Early stage of infection	0.473	0.158
Active phase of infection	0.473	0.158
Late of recurrent stage of infection	0.473	0.158
Past infection	0.257	0.086
Recovery stage of infection	0.129	0.043
Cost	0.086	0.257
Accessibility	0.473	0.158
Usability	0.257	0.086
Equipment	0.257	0.086
Education	0.257	0.086
Sensitivity	0.386	0.129
Specificity	0.386	0.129
False positivity	0.158	0.473
False negativity	0.158	0.473

**Table 3:** Weighted normalized data of the SARS-CoV-2 diagnostic tests.

Criteria	Importance weight	Max/Min	Naso/oropharyngeal swab PCR	Cell culture	CoV-19 antigen detection	CoV-19 antibody IgM	CoV-19 antibody IgG	Chest X-ray	Chest CT
No symptom-window period	VH	Max	0.47	0.47	0.47	0.16	0.16	0.16	0.32
Early stage	VH	Max	0.47	0.47	0.47	0.47	0.16	0.16	0.47
Active phase	VH	Max	0.47	0.47	0.47	0.47	0.47	0.16	0.47
Recurrent stage	VH	Max	0.16	0.16	0.16	0.16	0.47	0.32	0.47
Past infection	M	Max	0.09	0.09	0.09	0.09	0.26	0.17	0.26
Recovery stage	L	Max	0.04	0.04	0.04	0.09	0.13	0.04	0.13
Cost	M	Min	0.09	0.09	0.26	0.09	0.09	0.09	0.09
Accessibility	VH	Max	0.47	0.16	0.16	0.47	0.47	0.47	0.47
Usability	M	Max	0.26	0.09	0.26	0.26	0.26	0.26	0.26
Equipment	M	Max	0.17	0.09	0.26	0.09	0.09	0.09	0.26
Education	M	Max	0.17	0.09	0.09	0.26	0.26	0.17	0.26
Sensitivity	H	Max	0.13	0.39	0.13	0.13	0.13	0.26	0.39
Specificity	H	Max	0.39	0.39	0.13	0.26	0.26	0.26	0.39
False positivity	VH	Min	0.16	0.16	0.47	0.47	0.47	0.47	0.47
False negativity	VH	Min	0.47	0.16	0.47	0.47	0.47	0.47	0.47

VH: very high; H: high; M: medium; L: low.

**Table 4:** Complete ranking of SARS-CoV-2 diagnostic tests with fuzzy PROMETHEE.

Complete ranking	Diagnostic tests	Positive outranking flow (*ɸ*^+^)	Negative outranking flow (*ɸ*^−^)	Net flow (*ɸ*^net^)
1	Chest CT	0.0666	0.0109	0.0557
2	Naso/oropharyngeal swab PCR	0.0478	0.0220	0.0258
3	Cell culture	0.0635	0.0537	0.0098
4	CoV-19 antibody IgG	0.0384	0.0429	-0.0045
5	CoV-19 antibody IgM	0.0228	0.0391	-0.0163
6	CoV-19 antigen detection	0.0277	0.0543	-0.0267
7	Chest X-ray	0.0176	0.0615	-0.0439

**Table 5:** The relative closeness to positive ideal solution with the ranking of the SARS-CoV-2 diagnostic tests.

Ranking	Alternatives	*R*_*i*_
1	Chest CT	0.6403
2	Naso/oropharyngeal swab PCR	0.5865
3	Cell culture	0.5718
4	CoV-19 antibody IgG	0.4815
5	CoV-19 antibody IgM	0.4638
6	CoV-19 antigen detection	0.4460
7	Chest X-ray	0.3815

## Data Availability

The data used to support the findings of this study are included within the article.
